# Quantifying the checks and balances of collaborative governance systems for adaptive carnivore management

**DOI:** 10.1111/1365-2664.14113

**Published:** 2022-01-28

**Authors:** Jeremy J. Cusack, Erlend B. Nilsen, Markus F. Israelsen, Henrik Andrén, Matthew Grainger, John D. C. Linnell, John Odden, Nils Bunnefeld

**Affiliations:** ^1^ Centro de Modelación y Monitoreo de Ecosistemas Universidad Mayor Santiago Chile; ^2^ Biological and Environmental Sciences University of Stirling Stirling UK; ^3^ Norwegian Institute for Nature Research Trondheim Norway; ^4^ Grimsö Wildlife Research Station, Department of Ecology Swedish University of Agricultural Sciences Riddarhyttan Sweden; ^5^ Norwegian Institute for Nature Research Oslo Norway

**Keywords:** collaborative, decision‐making, harvest, lynx, Norway, population forecast, quota, stakeholder

## Abstract

1. Recovering or threatened carnivore populations are often harvested to minimise their impact on human activities, such as livestock farming or game hunting. Increasingly, harvest quota decisions involve a set of scientific, administrative and political institutions operating at national and sub‐national levels whose interactions and collective decision‐making aim to increase the legitimacy of management and ensure population targets are met. In practice, however, assessments of how quota decisions change between these different actors and what consequences these changes have on population trends are rare.
2. We combine a state‐space population modelling approach with an analysis of quota decisions taken at both regional and national levels between 2007 and 2018 to build a set of decision‐making models that together predict annual harvest quota values for Eurasian lynx (*Lynx lynx*) in Norway.
3. We reveal a tendency for administrative decision‐makers to compensate for consistent quota increases by political actors, particularly when the lynx population size estimate is above the regional target. Using population forecasts based on the ensemble of decision‐making models, we show that such buffering of political biases ensures lynx population size remains close to regional and national targets in the long term.
4. Our results go beyond the usual qualitative assessment of collaborative governance systems for carnivore management, revealing a system of checks and balances that, in the case of lynx in Norway, ensures both multi‐stakeholder participation and sustainable harvest quotas. Nevertheless, we highlight important inter‐regional differences in decision‐making and population forecasts, the socio‐ecological drivers of which need to be better understood to prevent future population declines.
5. *Synthesis and applications*. Our work analyses the sequence of decisions leading to yearly quotas for lynx harvest in Norway, highlighting the collaborative and structural processes that together shape harvest sustainability. In doing so, we provide a predictive framework to evaluate participatory decision‐making processes in wildlife management, paving the way for scientists and decision‐makers to collaborate more widely in identifying where decision biases might lie and how institutional arrangements can be optimised to minimise them. We emphasise, however, that this is only possible if wildlife management decisions are documented and transparent.

## Graphical abstract

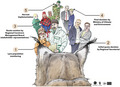

## 1 INTRODUCTION

The adaptive management of wildlife populations is an essential component of the interaction between biodiversity and human societies. Management can promote the conservation of threatened species in human‐dominated landscapes (Chapron et al., 2014; Karanth & DeFries, 2010), sustain economic, cultural and recreational human activities that rely on the extractive use of wild populations (Di Minin et al., 2019; Fischer et al., 2013), or minimise negative interactions that arise when wildlife affects, or is perceived to affect, human livelihoods (de Boon et al., 2021; Raithel et al., 2017; Redpath et al., 2013). In theory, decisions taken in the context of wildlife management aim to achieve one or more stated goal, such as protect threatened species, promote the sustainable use of harvested populations or reduce negative interactions between wildlife and humans. In many cases, poor management decisions can lead to the over‐exploitation or over‐abundance of wildlife populations (Bulte, 2001; Fryxell et al., 2010), either of which can affect human livelihoods and well‐being both locally and globally (Díaz et al., 2019). Assessing and understanding the factors that can influence the robustness of decision‐making is therefore of vital importance to ensuring effective and sustainable wildlife management and species survival (Polasky et al., 2011).

Management decisions relating to the harvest of large carnivore species pose a particular challenge owing to their economic and political significance (Artelle et al., 2018a, 2018b; Darimont et al., 2018; van Eeden et al., 2018), and the need to balance the interests of those promoting the harvest versus the protection of wild populations (Lute et al., 2020). This is especially the case when harvest is used as a tool to mitigate the negative impacts that a carnivore species of conservation concern can have on local human livelihoods, such as livestock depredation or competition with recreational hunting (van Eeden et al., 2018). Indeed, such scenarios often elicit strong responses from stakeholder groups with differing views on the value of lethal control, including, for example, wildlife conservationists, local communities and their political representatives (Redpath et al., 2013, 2017). In response to this, stakeholder co‐participation in management decisions—such as those surrounding quota values—is often promoted as a means to minimise conflict and increase both the effectiveness and acceptability of population control measures (Cusack et al., 2020; Mitchel et al., 2018; Pellikka & Sandström, 2011; Sandström & Lundmark, 2016).

Collaborative governance systems, whereby a range of actors participate in decision‐making (Emerson & Nabatchi, 2015; Hansson‐Forman et al., 2018; Sandström et al., 2009), are becoming increasingly common in the management of large carnivore populations worldwide (Curveira‐Santos et al., 2020; Lute et al., 2020; Redpath et al., 2017; Sandström et al., 2018; Treves et al., 2009). Such governance systems are typically characterised by a set of administrative and political institutions whose interactions and collective decision‐making—which may span local, regional and national scales in the case of decentralised governance—aim to increase the legitimacy of management and ensure population targets are met (Pellikka & Sandström, 2011; Risvoll et al., 2016; Sandström & Lundmark, 2016). Examples of collaborative governance systems associated with lethal control of carnivores include those involved in the development and implementation of harvest quotas for black bears *Ursus americanus* and cougars *Puma concolor* in both the United States and Canada (Artelle et al., 2018a), as well as for brown bears *Ursus arctos*, Eurasian lynx *Lynx lynx*, grey wolves *Canis lupus* and wolverines *Gulo gulo* in a range of European countries (de Boon et al., 2021). Furthermore, collaborative systems involving local communities are increasingly seen as key to ensuring the sustainability of trophy hunting activities targeted at large carnivores in both Africa and central Asia (Ullah & Kim, 2020).

Inherent to the functioning of collaborative governance is a careful balance between political pressures and the decision‐making process of specialised administrative entities whose role it is to evaluate and implement management actions based on scientific evidence (Artelle et al., 2018b; Fuller et al., 2020; Lute et al., 2014). Yet, the dynamic nature of this balancing act, including the relationship between complex decision‐making processes and their consequences for large carnivore management outcomes at national and subnational levels, is very rarely quantified, with the vast majority of assessments of collaborative governance systems relying on qualitative evaluations of stakeholder interactions and perceptions (de Boon et al., 2021; Jacobsen & Linnell, 2016; Sjölander‐Lindqvist et al., 2020). It is also absent from existing quantitative models of natural resource management, such as management strategy evaluation, which often replace complex decision‐making processes with single decisions based on simplified metrics (Cusack et al., 2020). Such approaches could hugely benefit from integrating more realistic decision‐making processes reflecting complex collaborative processes.

In this study, we quantify the set of decision‐making processes that lead to annual harvest quota values for Eurasian lynx (hereafter, lynx) in Norway. Lynx harvesting in Norway follows a quota‐regulated approach with a goal to maintain the population at a stable level (Linnell et al., 2010; Nilsen et al., 2012). The current national management goal of 65 lynx family groups (i.e. annual reproductions) was politically set by parliament in 2004, to be divided between eight management regions, each with a specific goal representing a proportion of the overall national target. Under this approach, regional decisions regarding lynx harvest quotas consist of a multi‐step process, starting with an initial proposal by the regional Secretariat hosted by the Office of the County Governor (hereafter, Secretariat), followed by a revision by a politically appointed Regional Carnivore Management Board (RCMB), a stakeholder appeal process, and a final decision by the Ministry of Climate and Environment (MCE; Andrén et al., 2020; Risvoll et al., 2016; Sandström et al., 2009; Sjölander‐Lindqvist et al., 2020). Although both regional and national targets aim to balance human interests and population viability, there is little ecological evidence to support them, with a recent study suggesting that the harvesting regime in Norway would be unsustainable without immigration from the larger Swedish population (Mills et al., 2018). Moreover, like in many large carnivore management systems, assessments of lynx harvesting in Norway have so far largely focused on the relationship between population predictions and final quota decisions (Andrén et al., 2020; Bischof et al., 2012; Nilsen et al., 2012). Consequently, the influence of the different decision‐making stages and the key interaction between administrative and political actors in shaping quota outcomes has not been analysed.

To address this gap, we combine a unique dataset of lynx quota decisions collected over the period from 2007 to 2018 for seven of the eight carnivore management regions with theoretically derived optimal quotas reflecting unbiased decision‐making. Such an approach enables us to evaluate inherent biases at each stage of the decision‐making process by comparing observed quotas with quota decisions that should have been taken to maximise the probability of achieving the population target. We then build an ensemble of models that relate successive changes in quota by the Secretariat, RCMB and the MCE, as well as the number of appeals, to a measure of management effectiveness that reflects how far the lynx population prediction for the current year is from the regional target. Using this model ensemble, we assess the ability of the quota decision‐making process to stabilise lynx population dynamics and achieve regional as well as national population targets in the long term.

## 2 MATERIALS AND METHODS

### 2.1 Study area

**FIGURE 1:**
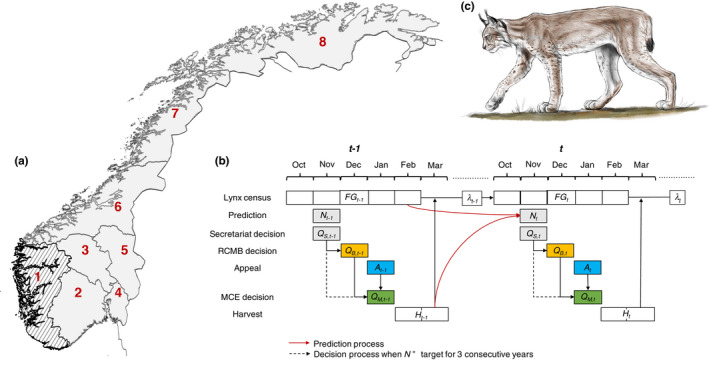
Map of carnivore management regions (a) and relative timings of census estimates (FG), population predictions (N), quota decisions (Q), appeal (A) and harvesting (H) processes (b) for Eurasian lynx in Norway (c). λ represents the growth of the lynx population between time steps after the effect of harvest on lynx abundance. The quota decision steps include an initial suggestion by the regional secretariat (Q_S_) based on lynx abundance at *t* − 1, followed by revision by the regional carnivore management board (RCMB; Q_B_). An appeal process takes place before the final quota decision is taken by the Norwegian Ministry for Climate and Environment (MCE; Q_M_). Note that decision power is removed from the RCMB if the estimated size of the lynx population is below target for three consecutive years. The shaded area in (a) represents management region 1, which is not included in the study area because the regional target is zero. Lynx illustration by Mattis Jayme van Dalum

The study area encompasses seven of the eight carnivore management regions in Norway (Figure 1a), which together cover approximately 273,000 km^2^. Management region 1 was excluded from the study since it has a population target of zero lynx family groups. Regions 2–8 are composed of alpine and boreal vegetation zones (Esseen et al., 1997), the former dominated by mountain birch *Betula pubescens* forests and the latter by Norway spruce *Picea abies* and Scots pine *Pinus sylvestris*. Most parts of the boreal forest are intensively managed for pulp and timber, which creates a mosaic of even‐aged forest stands. The proportion of agricultural land is generally low within the study area but increases towards the south.

Lynx in Norway occur in a multi‐use landscape alongside a variety of different human activities (Swenson & Andrén, 2005). In particular, management regions 7 and 8, as well as the northern and eastern parts of region 6, correspond to the reindeer husbandry area, in which the indigenous Sámi herd semi‐domestic, free‐ranging reindeer *Rangifer tarandus*. The latter are the primary prey of lynx in these regions, an impact that continues to sustain a significant conflict between lynx conservation and reindeer husbandry practices by the Sámi (Mattisson et al., 2011). Lynx predation on sheep occurs throughout the study area (Odden et al., 2008), while in the southern management regions, lynx predation on roe deer *Capreolus capreolus* is also a source of conflict between lynx conservation and local hunting activities (Odden et al., 2006).

### 2.2 Lynx population model

Lynx monitoring in Norway follows a common methodology across all carnivore management regions based on non‐replicated counts of annual reproductions, which since 2002 has been coordinated at a national level by the National Large Predator Monitoring Program (Andrén et al., 2020; Nilsen et al., 2012). Lynx census efforts are carried out every winter between the months of November and February. Importantly, lynx quotas for a given winter *t* are set before estimates of lynx population size are available for that same winter (*N*_*t*_). Prior to 2012, quota decisions were based on the lynx count recorded for the previous winter (*N*_*t*‐1_) (i.e. count‐based strategy). Since 2012, a state‐space population model has been made available to the regional Secretariats (Buckland et al., 2004; Nilsen et al., 2011), which enables estimation of lynx population size at *t* based on the time series of observed number of reproductive females (hereafter, family groups) and harvest bags collected up until *t* − 1. Using this model, we generated predictions of the pre‐harvest lynx population size for each region and year *t* between 2012 and 2018, representing the period during which the model was available to the regional Secretariats (i.e. model‐based strategy). Details of model structure, fitting and evaluation are provided in Appendix S1.

### 2.3 Lynx quota decision‐making process and data

The timeline for lynx monitoring, quota‐setting and quota implementation in Norway is shown in Figure 1b (Andrén et al., 2020; Risvoll et al., 2016). In this study, we focus on three key stages of decision‐making. The first stage relates to the initial quota decision in November of winter *t* by the professional administration in the Secretariat (hereafter, Secretariat quota) based on lynx monitoring results from winter *t* − 1 or a model prediction for *t* (Andrén et al., 2020; Nilsen et al., 2011). This initial quota suggestion is passed on in December of winter *t* to a Regional Carnivore Management Board (RCMB), which is made up of local‐level politicians appointed by the ministry at the national level. The RCMB can revise the quota depending on the input of board members and the interests they represent (hereafter, RCMB quota; Risvoll et al., 2016). The resulting quota then undergoes an appeal process, whereby groups with stakes in lynx management (e.g. reindeer herders, sheep farmers, hunters and conservationists) can seek changes to the decision. The quota proposed by the RCMB and the corresponding appeals are reviewed by the MCE, which decides in January on a final quota to be implemented during the months of February and March of winter *t* (hereafter, MCE quota). Importantly, if the predicted lynx population size is below the regional population target for three consecutive years, the quota decision power of the RCMB is removed until the population increases above the target. In such cases, quota decisions go directly from the Secretariat to the appeal process. In all cases, the MCE has authority on the final quota decision.

We extracted quota values resulting from each of the decision‐making stages (i.e. Secretariat, RCMB and MCE) as well as the number of appeals made from both regional and national sources. The quota suggestion by the Secretariat and the decision made by the RCMB were both extracted from publicly available meeting documents uploaded to the respective websites of each region. The number of appeals and final quota decision made by the MCE were extracted from documents made available publicly on the Norwegian government homepage (https://miljovedtak.no/). For years for which online documents were not available, the County Governor of each management region was contacted to obtain meeting documents relating to appeals and MCE decisions. Complete quota decision and appeal data were only available from 2007 onwards.

### 2.4 Optimal quota decisions

To serve as a general evaluation of observed quota decisions, we derived, for each region *k* and winter *t*, the optimal decision *Q*(opt)_*t,k*_ that should have been taken to maximise chances of reaching the regional target (*L*_*k*_) at *t* + 1. For a given region, this objective is expressed as:

(1) $$N_{t+1,k}=L_{k},$$

in which *N*_*t*+1*,k*_ represents the lynx population size in region *k* at *t* + 1. Following Andrén et al. (2020), we assume that:

(2) $$N_{t+1,k}=\left(N_{t,k}-Q_{t,k}\right)*{\bar{\lambda }}_{k},$$

in which *N*_*t,k*_ and *Q*_*t,k*_ represent the estimated lynx population size and harvest quota for region *k* at winter *t*, respectively, and ${\bar{\lambda }}_{k}$ denotes the region‐specific mean population growth rate. Combining Equations (1) and (2) yields:

(3) $$L_{k}=\left(N_{t,k}-Q{\left(\mathrm{opt}\right)}_{t,k}\right)*{\bar{\lambda }}_{k}.$$

Re‐arranging, we obtain a model for the optimal quota decision (optimal quota model):

(4) $$Q{\left(\mathrm{opt}\right)}_{t,k}=N_{t,k}-\frac{L_{k}}{{\bar{\lambda }}_{k}}.$$

Values of $Q{\left(\mathrm{opt}\right)}_{t,k}$ that were < 0 were set to 0.

### 2.5 Comparison of observed and optimal quota values

We modelled observed quota as a function of the interaction between decision stage (Secretariat, RCMB and MCE) and region using a generalised linear mixed‐effect model (GLMM) with a negative binomial error structure, year as a random intercept and log(*N*_*t*_) as an offset. The latter was included to correct for varying lynx population size, in effect converting the response variable into a quota rate, which can be compared across management regions. In this model, factor levels representing the Secretariat decision and Region 2 were included as reference values against which the effects of other factor levels were evaluated. We then fit a second GLMM, which this time included the optimal quota decision as reference level for the decision stage factor (i.e. Optimal, Secretariat, RCMB and MCE), to evaluate the extent to which observed quota decisions deviated from the corresponding optimal decision.

In the case of the Secretariat decision, we further assessed how the difference between observed and optimal quotas for each region *k* varied as a function of a measure of management effectiveness defined as the population–target ratio (PTR) = $\frac{N_{t}}{L_{k}}$. This measure is equal to 1 when *N*_*t*_ = *L*_*k*_, <1 when *N*_*t*_ < *L*_*k*_, and >1 when *N*_*t*_ > *L*_*k*_. Our null expectation of unbiased decision‐making was that the Secretariat quota decision would deviate as little as possible from optimal and for the difference between observed and optimal values to remain constant across values of PTR. In other words, observed decisions are always optimal regardless of population abundance.

### 2.6 Modelling changes in quota across decision‐making stages

We used a combination of linear regression models to model successive changes in quota value between the initial Secretariat decision and the final MCE decision as a function of the PTR. We used the latter value as a measure of management effectiveness that we assumed was understood and considered at all stages of decision‐making.

In the first instance, we modelled the initial Secretariat decision at time *t* as a function of the interaction between the PTR and a categorical variable reflecting the management region. This model assumes that the manager adjusts quota decisions based on how far the predicted lynx population size at *t* is from the regional target, but that this process varies predictably depending on the region (Andrén et al., 2020). We chose to implement a linear mixed model (LMM) as preliminary analyses indicated that treating the Secretariat quota as a count and fitting a GLMM with a negative binomial error structure would result in strictly positive intercept values, reflecting the unrealistic setting of positive quota values at a value of *N*_*t*_ equal to 0.

Decision stages relating to the RCMB, the appeal process and the MCE were each modelled using a two‐step approach akin to a hurdle model. The first step consisted of a binomial GLMM for which the response was a binary variable reflecting the presence/absence of a change of quota in the case of the RCMB and MCE stages, or the presence/absence of at least one appeal. Predictor variables for the RCMB and appeal stage models consisted of the interaction between the PTR and region, while for the MCE stage, the number of appeals, the PTR and region were included as additive effects. The second step in our approach considered only instances in which a quota change or at least one appeal was recorded. For the RCMB and appeal stages, this took the form of a negative binomial GLMM for which the response variable was quota increase (only positive changes were recorded) and number of appeals, respectively, and the predictor variables were the interacting effects of the PTR and region. For the MCE stage, we modelled quota change as a function of the number of appeals and the PTR, both of which interacted independently with region, using an LMM to account for both negative and positive changes in quota.

In all models, year was included as a random intercept to account for the temporal dependency between quota decisions and appeals carried out in consecutive years. Model selection was carried out by ranking candidate models based on the AICc value. We combined all variables present in models within 4 delta AICc into a top model, which we then used to make inferences and subsequent predictions. All analyses were carried out R using packages lme4 (Bates et al., 2015) and glmmTMB (Brooks et al., 2017).

### 2.7 Population forecasting

We used the ensemble of decision‐making models selected in the previous section to predict, for each management region, lynx population dynamics for the years 2019–2030. Stochasticity was included in each of 1000 iterations by sampling the annual growth rate from a normal distribution with mean ${\bar{\lambda }}_{k}$ and associated standard deviation $\mathrm{sd}\left({\bar{\lambda }}_{k}\right)$. Here, ${\bar{\lambda }}_{k}$ is the mean growth rate over the period 1996–2018, as would have been estimated by regional Secretariats in 2018 (Appendix S2, Table S2‐2). All other component parameters of decision stage models were represented by their estimated mean value. Importantly, our forecasts assume that the harvest quota is implemented perfectly, enabling us to assess the effect of decision processes without the confounding effect of implementation uncertainty. We summed predictions across regions to obtain a forecasted trend at the national level.

## 3 RESULTS

### 3.1 Lynx population size estimates

We predicted values of *N*_*t*_ for each year between 2007 and 2018, using the count‐based strategy prior to 2012 and the model‐based strategy from 2012. Comparison of predicted and observed values of *N*_*t*_ indicated good predictive power for both count and model‐based approaches (see Appendix S2, Figures S2‐1 and S2‐2).

### 3.2 Lynx quota decisions

We analysed a total of 84 quota decision processes—each combining successive Secretariat, RCMB and MCE decisions—collected between 2007 and 2018 across the seven management regions (Appendix S2, Figure S2–S3). Data from 2007 and 2008 in Region 4 were excluded from our analysis due to missing quota values for two of the decision‐making stages. Of the remaining 82 decision processes, 19 reflected processes in which decision‐making power was removed from the RCMB following three consecutive years below the population target (i.e. 23.2% of all decision processes with only two decisions instead of three), resulting in a total of 227 decisions analysed.

**FIGURE 2:**
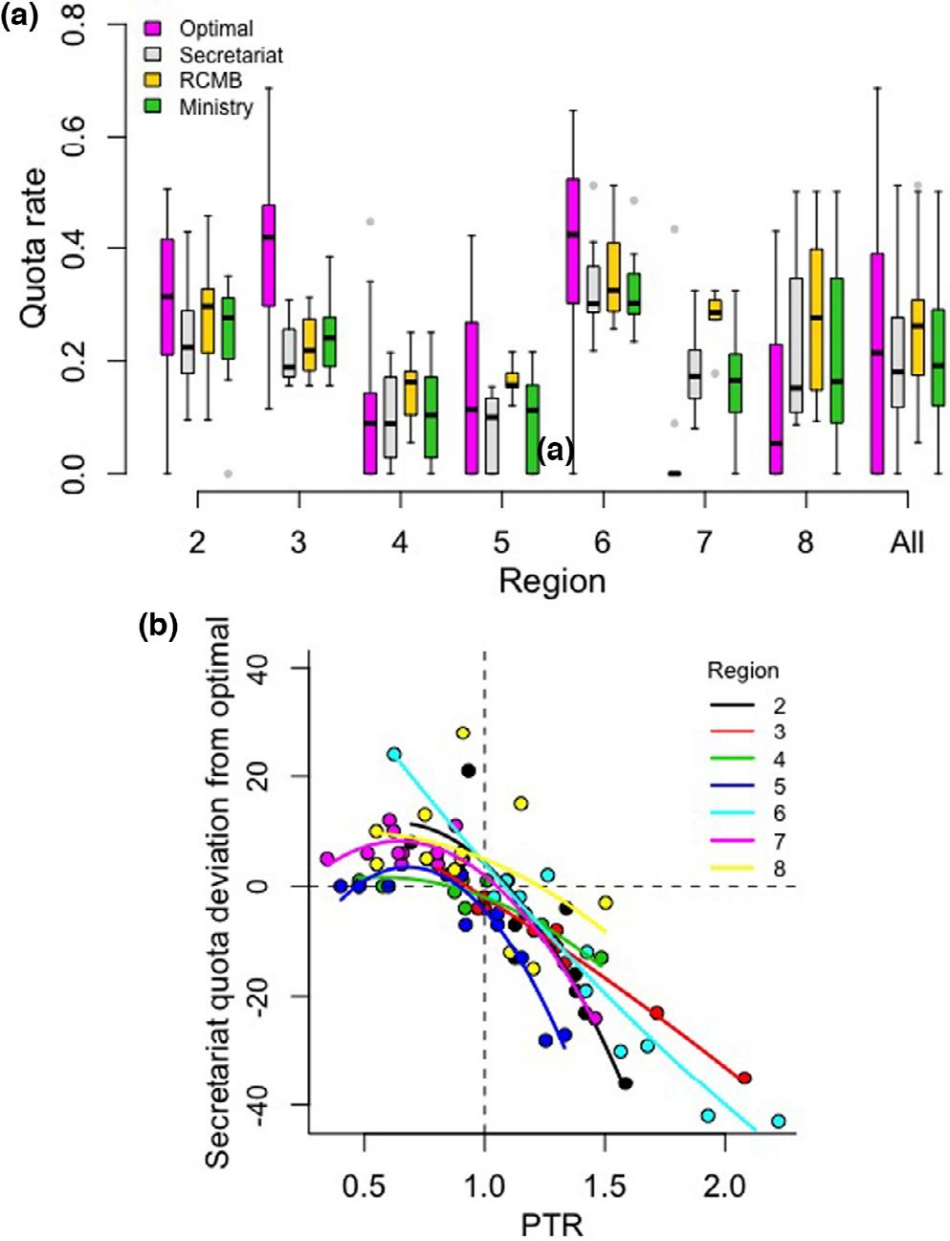
Optimal and observed quota rates for Norwegian lynx in management regions 2–8 (a) and relationship between the secretariat quota deviation from optimal and a measure of management effectiveness, the population to target ratio (PTR) (b). The PTR is equal to 1 when lynx population size at time *t* is equal to the population target, <1 when population size is below the target and >1 when population size is above the target. In (a), the optimal quota rate is based on the theoretical model defined in the materials and methods (see Equation 4) while observed values are the result of decisions taken by the secretariat, the regional carnivore management board (RCMB) and the Norwegian Ministry of Climate and Environment (MCE). Lines in (b) represent predictions from a fitted linear mixed effects model with PTR^2^ and region as interacting effects and year as a random intercept. Horizontal and vertical dashed lines in (b) denote cases when the secretariat quota equals the optimal quota and when the estimated lynx population size at t equals the regional population target, respectively

There was very strong evidence for differing quota rates across regions (likelihood‐ratio test based on nested negative binomial Generalised Linear Mixed Models with year as random effect: *χ* ^2^ = 156.0, *df* = 6, *p* < 0.001), with regions 6 and 4 showing the highest and lowest values on average (Figure 2a). Differences in quota rate were also moderate across decision stages (*χ* ^2^ = 7.1, *df* = 2, *p* < 0.05), with quota rates resulting from the RCMB tending to be higher than those from either the Secretariat or MCE in all regions except region 3, where the MCE quota rate was highest on average. The percentages of Secretariat decisions that were decreased, unchanged or increased by the RCMB were 0, 50.8 and 49.2% (*n* = 63), respectively, whereas the percentages of either Secretariat or RCMB decisions that were decreased, unchanged or increased by the MCE were 11.0, 81.7 and 7.3% (*n* = 82), respectively.

In contrast to differences among observed quota rates, the difference among optimal and observed quota rates varied depending on the interaction between decision stage and region (*χ* ^2^ = 62.2, *df* = 18, *p* < 0.001). More specifically, the Secretariat quota rate tended to be lower than optimal in regions 2 to 6 and higher in regions 7 and 8 (Figure 2a). This was reflected in the relationship between Secretariat quota deviation from optimal and the PTR, which was best modelled as an interaction between region and the quadratic term PTR^2^. According to this model, the Secretariat quota decision tended to be closer to optimal when *N*_*t*_ was equal to or below the regional target (i.e. PTR ≤1) and below when *N*_*t*_ was above the regional target (Figure 2b).

### 3.3 Modelling changes in quota

**FIGURE 3:**
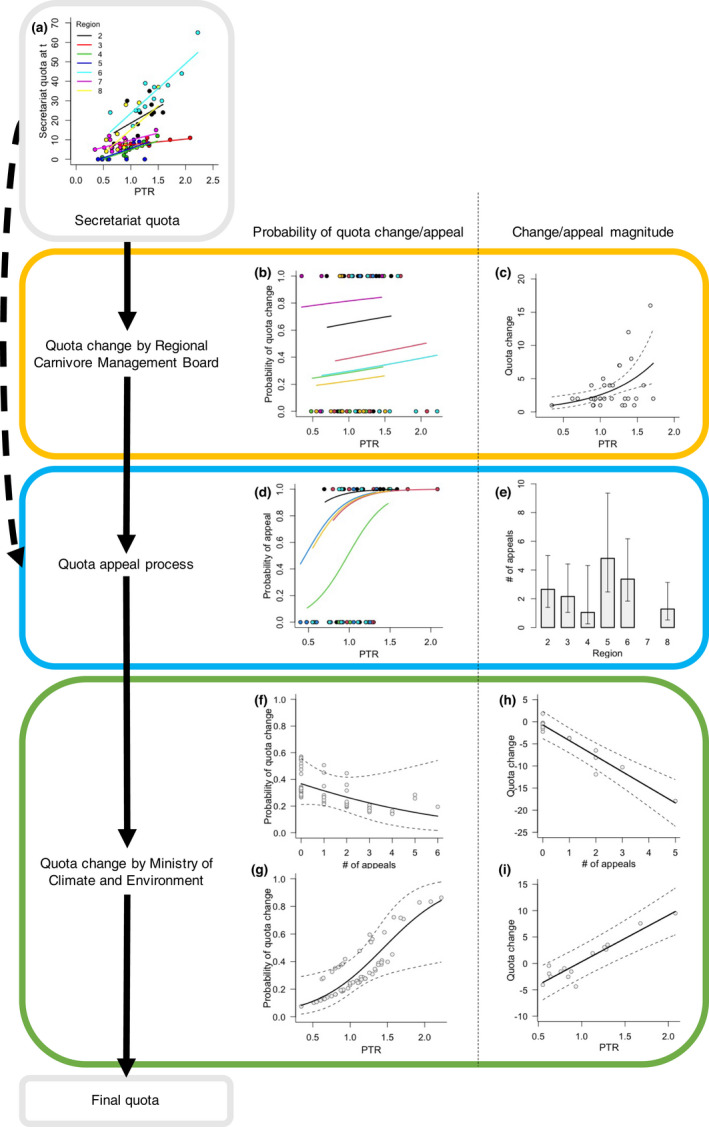
Summary of decision‐making processes occurring between the initial lynx quota suggestion by the regional secretariat (a) and the final quota, including the revision by the regional carnivore management board (b and c), quota appeals (d and e), and the final decision by the Norwegian Ministry of Climate and Environment (f, g, h and i). The RCMB, appeal and MCE stages each consist of a two‐step process whereby the probability of quota change or appeal and the magnitude of quota change or number of appeals are estimated successively. In all cases, bars and lines with corresponding error brackets and dashed lines represent predictions and associated confidence intervals from fitted models described in Table S2, respectively. Note that in (f), (g), (h) and (i) grey dots represent partial residuals. The full and dashed arrows linking decision stages represent process in the presence and absence of a decision by the RCMB, respectively. The PTR is equal to 1 when lynx population size at time *t* is equal to the population target, <1 when population size is below the target and >1 when population size is above the target

Model selection outputs revealed that the Secretariat quota decision was positively influenced by the PTR and that the slope of this effect varied across regions (Figure 3a; Appendix S2, Table S2‐1). The probability that the RCMB would seek a quota change following the initial proposal by the Secretariat depended on both the PTR and the region (Figure 3b), with regions 5 and 8 showing the highest (average predicted probability of 1 as a function of the PTR) and lowest probability ranges (average predicted probability of 0.22 [95% CIs 0.06–0.58]), respectively. When a change did occur, its magnitude was positively related to the PTR, a relationship that was common to all management regions (Figure 3c).

Overall, appeals were more likely to occur with increasing PTR, although the shape of this relationship depended on the region (Figure 3d). Appeals were recorded every year for region 6, resulting in predicted probabilities of 1 (Appendix S2, Figure S2‐4). In contrast, no appeals were recorded for region 7 leading to predicted probabilities of 0. When appeals did occur, their number was best predicted by the management region (Figure 3e), with regions 4 and 5 being characterised by the lowest (1.10, [0.26–4.31]) and highest (4.81 [2.48–9.35]) predicted number of appeals, respectively. Lastly, the MCE was more likely to modify the quota received from either the Secretariat or the RCMB when the number of appeals was low (Figure 3f) and the PTR value was high (Figure 3g). Similarly, the magnitude and direction of the resulting change was negatively influenced by the number of appeals received (Figure 3h) and positively related to the PTR (Figure 3i).

### 3.4 Population forecasting

**FIGURE 4:**
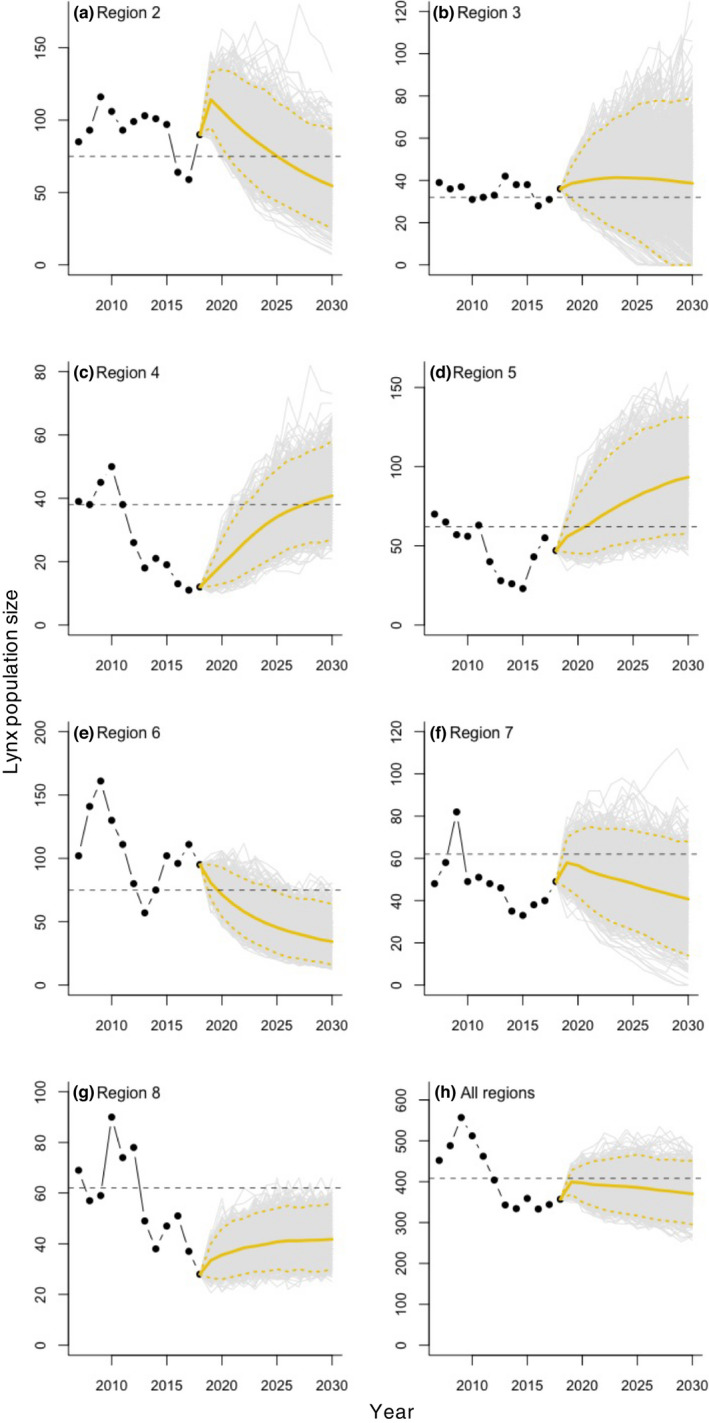
Lynx population forecasts for the years 2019–2030 based on the ensemble of decision‐making models characterising quota decisions, including the initial proposal by the secretariat, the revision by the regional carnivore management board, the appeal process, and the final decision by the Ministry of Climate and Environment. Panels (a) to (g) show forecasts for management regions 2 to 8, respectively, whilst panel (h) shows the national forecast. Black dots represent estimated lynx population sizes for the years 2007–2018 as derived from a state‐space population model applied to lynx census and harvest data collected between 1996 and 2018. The full yellow line represents the mean population trend across 1000 iterations and the dashed lines denote the associated 95% confidence intervals. The horizontal dashed line marks the population target

We used the ensemble of decision‐making models governing quota setting by the Secretariat, quota changes by the RCMB and MCE, and the number of appeals made to predict, for each region and for Norway as a whole, lynx population dynamics for the years 2019–2030. Such a forecast acts as a valuable evaluation of the ability of the entire decision‐making process to maintain lynx population size on target. Although long‐term population predictions for all regions generally overlapped with the stated target, trend direction varied across regions (Figure 4). Regions 2, 3, 6 and 7 exhibited negative population trends that over time resulted in lynx numbers that were below the population target (Figure 4a, b, d and e). In contrast, regions 4 and 8 showed positive trends that enabled long‐term recovery and stabilisation of lynx numbers close to the population target (Figure 4c and g). Lastly, the forecast for region 5 indicated growth beyond the population target (Figure 4f). This heterogeneity in long‐term forecasts at a regional level resulted in predictions at a national level that, although overlapping with the national target, indicated a weak but consistent population decline over time (Figure 4h).

## 4 DISCUSSION

Our analysis of lynx quota decisions by administrative and political entities in Norway and associated population forecasts reveal a system of checks and balances that, overall, successfully maintains lynx population size close to the national target despite strong opposing pressures from conservation, farming and hunting interests at a regional level (Jacobsen & Linnell, 2016; Linnell et al., 2010). These pressures manifest themselves at key stages in the decision‐making process, namely the quota revision by the politically appointed RCMB and the appeal process occurring prior to the final decision by the MCE. RCMBs, in particular, are often highly biased in their representativeness towards the interests of farmers and hunters (Risvoll et al., 2016), resulting in a quota revision that is consistently upwards when it occurs. This is especially the case when the lynx population in the previous year is estimated to be above the regional target, reflecting a strong motivation to keep lynx numbers under control.

This tendency for the RCMB to increase quota values appears to be anticipated for by the regional Secretariats, which we find were more likely to bias their quota proposals downward from the theoretically optimal value when the lynx population estimates were above the regional target. This pro‐conservative behaviour did not occur when the lynx population estimates were below or equal to the regional targets, in which case the Secretariats' quotas tended to be closer to optimal. It is unlikely, however, that suboptimal decision‐making by the Secretariats aimed to compensate for a potential increase by the MCE, which also tended to occur at higher population to target ratios. This is because, in a first instance, quota changes by the MCE were relatively rare, only occurring for one in five decisions. Moreover, the MCE decisions to increase or decrease a quota were also mostly negatively influenced by the number of appeals received following the RCMB decision.

Our analysis highlights regional differences in quota decision processes and their ability to maintain stable lynx populations over time. In particular, population trends were predicted to be positive in regions 4, 5 and 8, areas in which sharp declines were recorded between 2010 and 2018. Such predicted recoveries could be driven by lower PTR values in these regions, resulting in smaller quota increases by the RCMB and, consequently, final quota decisions that are closer to optimal. In the case of region 8, such a recovery still resulted in population predictions that were below the regional target, a long‐term outcome also shown by regions 6 and 7. Although it remains unclear which aspect of the regional decision‐making process drives these downward trends, it is important to highlight that these northern regions are characterised by high numbers of lynx relative to southern regions (with the exception of region 2, which also showed a negative trend). This could result in a tendency to over‐compensate even when lynx numbers decrease below the population target. Fryxell et al. (2010) showed that delayed or over‐compensatory harvesting behaviour could lead to oscillating population trends, a pattern exhibited most strongly by regions 2 and 7 in the present analysis. Furthermore, higher lynx numbers may exacerbate ongoing conflicts between lynx conservation and both reindeer and sheep husbandry (Mattisson et al., 2011; Strand, 2021; Tveraa et al., 2014), which may lead to stronger control of lynx populations. Population declines in these regions likely underlie the weakly negative trend forecasted at the national level, emphasising the importance of understanding the links between socio‐ecological context, stakeholder interests and collaborative decision‐making outcomes.

Our work provides important insights into how interactions between the different actors involved in collaborative governance systems can buffer political influences on wildlife management decisions and lead to stable wildlife population trends (Darimont et al., 2018). In particular, our findings echo of the ‘tug of war’ concept used by Orach et al. (2020) to characterise the feedback mechanism between stakeholder decisions that they find stabilises European Union fisheries quotas by counterbalancing the influence of opposing interests. Importantly, they observe that such a mechanism can be beneficial to natural resource management, sometimes delaying or preventing stock collapse. In a similar way, buffering of the political influence of the RCMB and MCE by the Secretariat and the appeal process in the case of Norwegian lynx quotas may ensure population viability in the long‐term despite competing interests.

Although our approach represents a novel way to integrate population modelling and quantitative decision‐making analysis, it is important to acknowledge its scope and limitations. First, our state‐space model provides a relatively simple approximation of the factors driving lynx numbers, omitting by necessity processes such as compensatory or super‐additive responses to harvest, as well as emigration and immigration dynamics (Mills et al., 2018). Similarly, it does not account for the influence of illegal killing as an additional source of mortality, whose importance varies from region to region (Andrén et al., 2006, 2020). Second, our forecasting exercise considers harvest implementation to be perfect, an assumption that enables us to focus on evaluating the influence of harvest decisions on lynx population dynamics. However, we acknowledge that realised quotas for lynx in Norway, as well as for most other harvested carnivores, may not always fulfil stated quotas (Bischof et al., 2012). Lastly, the quota appeal data considered in this study did not include information regarding the stakeholder group to which the claimant belonged, which may mask potential conflicts of interest driving quota decisions. We recommend future studies seek to clarify in more detail the role of the appeal process in shaping final decisions by the MCE.

Quantitative assessments of decision‐making at the heart of large carnivore management are only possible when decisions at each stage of the process are transparent (Artelle et al., 2018a, 2018b; Fuller et al., 2020). As shown by the present study, such data transparency enables evaluations of management effectiveness to go beyond their usual focus on monitoring biases to encompass relations between stakeholder interests, including the consequences of individual decision strategies. In the case of Norwegian lynx, the effect of these decision biases on population management is at least partly tempered by the decentralised governance system as a whole. Yet, extending this conclusion to other collaborative governance systems remains challenging given the structural and implementation differences that exist across countries and species (de Boon et al., 2021; Sjölander‐Lindqvist et al., 2020). Indeed, no such quantitative analysis that we are aware of exists for other managed species, and our approach thus serves as a template for assessing the effectiveness of collaborative governance systems for species that are managed through harvesting. In particular, we urge scientists and decision‐makers to collaborate more widely in identifying where decision biases might lie and how institutional arrangements can be optimised to minimise them (Hartel et al., 2019; Redpath et al., 2017; Treves et al., 2017). Our approach may not only be beneficial for species whose populations are harvested to minimise conflict with human activities, but also for those species that are trophy hunted, an activity for which lack of transparency in decision‐making has contributed towards fuelling a debate over its value and legitimacy (Treves et al., 2019).

In summary, our work provides a predictive framework to evaluate participatory decision‐making processes in wildlife management (Travers et al., 2019). Key to this is the collection of both long‐term ecological and quota decision data, which together enable the parametrisation and integration of population and decision‐making models. Not only can this approach reveal the mechanisms underlying quota harvest decision processes, but it can also be used to generate more realistic predictions of wildlife population dynamics that account for biased human decisions. Such knowledge is key to ensuring wildlife population targets are met in the presence of competing stakeholder interests.

## CONFLICT OF INTEREST

The authors declare no conflict of interest.

## AUTHORS' CONTRIBUTIONS

J.J.C. carried out the modelling and wrote the manuscript; M.F.I. collected the quota decision data; E.B.N., H.A., J.D.C.L. and J.O. developed the population model; M.G. and N.B. assisted with data analysis. All authors contributed critically to the drafts and gave final approval for publication.

## Supporting information


Appendix S1

Appendix S2

## Data Availability

Data available via the Open Science Framework (OSF) Repository 10.17605/OSF.IO/FZ2CV (Cusack et al., 2021).

## References

[jpe14113-bib-0001] Andrén, H. , Linnell, J. D. , Liberg, O. , Andersen, R. , Danell, A. , Karlsson, J. , Odden, J. , Moa, P. F. , Ahlqvist, P. , Kvam, T. , Franzén, R. , & Segerström, P. (2006). Survival rates and causes of mortality in Eurasian lynx (*Lynx lynx*) in multi‐use landscapes. Biological Conservation, 131, 23–32.

[jpe14113-bib-0002] Andrén, H. , Thompson Hobbs, N. , Aronsson, M. , Brøseth, H. , Chapron, G. , Linnell, J. D. C. , Odden, J. , Persson, J. , & Nilsen, E. B. (2020). Harvest models of small populations of a large carnivore using Bayesian forecasting. Ecological Applications, 30, e02063. 31868951 10.1002/eap.2063 PMC7187313

[jpe14113-bib-0003] Artelle, K. A. , Reynolds, J. D. , Treves, A. , Walsh, J. C. , Paquet, P. C. , & Darimont, C. T. (2018a). Working constructively toward an improved north American approach to wildlife management. Science Advances, 4(10), eaav2571. 30306136 10.1126/sciadv.aav2571 PMC6170033

[jpe14113-bib-0004] Artelle, K. A. , Reynolds, J. D. , Treves, A. , Walsh, J. C. , Paquet, P. C. , & Darimont, C. T. (2018b). Hallmarks of science missing from north American wildlife management. Science Advances, 4(3), eaao0167. 29532032 10.1126/sciadv.aao0167 PMC5842039

[jpe14113-bib-0005] Bates, D. , Maechler, M. , Bolker, B. , & Walker, S. (2015). Fitting linear mixed‐effects models using lme4. Journal of Statistical Software, 67, 1–48.

[jpe14113-bib-0006] Bischof, R. , Nilsen, E. B. , Brøseth, H. , Männil, P. , Ozolinš, J. , & Linnell, J. D. C. (2012). Implementation uncertainty when using recreational hunting to manage carnivores. Journal of Applied Ecology, 49, 824–832. 23197878 10.1111/j.1365-2664.2012.02167.x PMC3504070

[jpe14113-bib-0007] Brooks, M. E. , Kristensen, K. , van Benthem, K. J. , Magnusson, A. , Berg, C. W. , Nielsen, A. , Skaug, H. J. , Machler, M. , & Bolker, B. (2017). glmmTMB balances speed and flexibility among packages for zero‐inflated generalized linear mixed modeling. The R Journal, 9, 378–400.

[jpe14113-bib-0008] Buckland, S. T. , Newman, K. B. , Thomas, L. , & Koesters, N. B. (2004). State‐space models for the dynamics of wild animal populations. Ecological Modelling, 171, 157–175.

[jpe14113-bib-0009] Bulte, E. H. (2001). Minimum viable populations and sluggish management. Environmental Conservation, 28, 191–193.

[jpe14113-bib-0010] Chapron, G. , Kaczensky, P. , Linnell, J. D. C. , von Arx, M. , Huber, D. , Andrén, H. , López‐Bao, J. V. , Adamec, M. , Álvares, F. , Anders, O. , Balčiauskas, L. , Balys, V. , Bedö, P. , Bego, F. , Blanco, J. C. , Breitenmoser, U. , Brøseth, H. , Bufka, L. , Bunikyte, R. , … Boitani, L. (2014). Recovery of large carnivores in Europe’s modern human‐dominated landscapes. Science, 346, 1517–1519. 25525247 10.1126/science.1257553

[jpe14113-bib-0011] Curveira‐Santos, G. , Sutherland, C. , Santos‐Reis, M. , & Swanepoel, L. H. (2020). Responses of carnivore assemblages to decentralized conservation approaches in a south African landscape. Journal of Applied Ecology, 58, 92–103. 10.1111/1365-2664.13726

[jpe14113-bib-0012] Cusack, J. J. , Duthie, A. B. , Minderman, J. , Jones, I. L. , Pozo, R. A. , Rakotonarivo, O. S. , Redpath, S. , & Bunnefeld, N. (2020). Integrating conflict, lobbying, and compliance to predict the sustainability of natural resource use. Ecology and Society, 25(2), 13.

[jpe14113-bib-0013] Cusack, J. J. , Nilsen, E. B. , Israelsen, M. F. , Andrén, H. , Grainger, M. , Linnell, J. D. C. , Odden, J. , & Bunnefeld, N. (2021). Data from: Quantifying the checks and balances of collaborative governance systems for adaptive carnivore management. Open Science Framework. 10.17605/OSF.IO/FZ2CV PMC9306889 35910004

[jpe14113-bib-0014] Darimont, C. T. , Paquet, P. C. , Treves, A. , Artelle, K. A. , & Chapron, G. (2018). Political populations of large carnivores. Conservation Biology, 32, 747–749. 29363171 10.1111/cobi.13065

[jpe14113-bib-0015] de Boon, A. , Sandström, C. , Arbieu, U. , Hansen, I. , Lehnen, L. , Marino, A. , Mari, P.‐M. , Risvoll, C. , Strand, G.‐H. , & Rønningen, K. (2021). Governing dual objectives within single policy mixes: An empirical analysis of large carnivore policies in six European countries. Journal of Environmental Policy & Planning, 23(4), 399–413.

[jpe14113-bib-0016] Di Minin, E. , Brooks, T. M. , Toivonen, T. , Butchart, S. H. M. , Heikinheimo, V. , Watson, J. E. M. , Burgess, N. D. , Challender, D. W. S. , Goettsch, B. , Jenkins, R. , & Moilanen, A. (2019). Identifying global centers of unsustainable commercial harvesting of species. Science Advances, 5, eaau2879. 30949571 10.1126/sciadv.aau2879 PMC6447386

[jpe14113-bib-0017] Díaz, S. , Settele, J. , Brondízio, E. S. , Ngo, H. T. , Agard, J. , Arneth, A. , Balvanera, P. , Brauman, K. A. , Butchart, S. H. M. , Chan, K. M. A. , Garibaldi, L. A. , Ichii, K. , Liu, J. , Subramanian, S. M. , Midgley, G. F. , Miloslavich, P. , Molnáe, Z. , Obura, D. , Pfaff, A. , & Polasky, S. (2019). Pervasive human‐driven decline of life on earth points to the need for transformative change. Science, 366(6471), eaax3100. 31831642 10.1126/science.aax3100

[jpe14113-bib-0018] Emerson, K. , & Nabatchi, T. (2015). Collaborative governance regimes. Georgetown University Press.

[jpe14113-bib-0019] Esseen, P. A. , Ehnström, B. , Ericson, L. , & Sjöberg, K. (1997). Boreal forests. Ecological Bulletins, 46, 16–47.

[jpe14113-bib-0020] Fischer, A. , Kereži, V. , Arroyo, B. , Mateos‐Delibes, M. , Tadie, D. , Lowassa, A. , Krange, O. , & Skogen, K. (2013). (De) legitimising hunting–discourses over the morality of hunting in Europe and eastern Africa. Land Use Policy, 32, 261–270.

[jpe14113-bib-0021] Fryxell, J. M. , Packer, C. , McCann, K. , Solberg, E. J. , & Sæther, B. E. (2010). Resource management cycles and the sustainability of harvested wildlife populations. Science, 328, 903–906. 20466934 10.1126/science.1185802

[jpe14113-bib-0022] Fuller, A. K. , Decker, D. J. , Schiavone, M. V. , & Forstchen, A. B. (2020). Ratcheting up rigor in wildlife management decision making. Wildlife Society Bulletin, 44, 29–41.

[jpe14113-bib-0023] Hansson‐Forman, K. , Reimerson, E. , Sjölander‐Lindqvist, A. , & Sandström, C. (2018). Governing large carnivores—Comparative insights from three different countries. Society & Natural Resources, 31, 837–852.

[jpe14113-bib-0024] Hartel, T. , Scheele, B. C. , Vanak, A. T. , Rozylowicz, L. , Linnell, J. D. C. , & Ritchie, E. G. (2019). Mainstreaming human and large carnivore coexistence through institutional collaboration. Conservation Biology, 33, 1256–1265. 30997704 10.1111/cobi.13334

[jpe14113-bib-0025] Jacobsen, K. S. , & Linnell, J. D. (2016). Perceptions of environmental justice and the conflict surrounding large carnivore management in Norway—Implications for conflict management. Biological Conservation, 203, 197–206.

[jpe14113-bib-0026] Karanth, K. K. , & DeFries, R. (2010). Conservation and management in human‐dominated landscapes: Case studies from India. Biological Conservation, 143, 2865–2964.

[jpe14113-bib-0027] Linnell, J. D. , Brøseth, H. , Odden, J. , & Nilsen, E. B. (2010). Sustainably harvesting a large carnivore? Development of Eurasian lynx populations in Norway during 160 years of shifting policy. Environmental Management, 45, 1142–1154. 20213233 10.1007/s00267-010-9455-9

[jpe14113-bib-0028] Lute, M. L. , Bump, A. , & Gore, M. L. (2014). Identity‐driven differences in stakeholder concerns about hunting wolves. PLoS One, 9, e114460. 25464276 10.1371/journal.pone.0114460 PMC4252115

[jpe14113-bib-0029] Lute, M. L. , Carter, N. H. , López‐Bao, J. V. , & Linnell, J. D. (2020). Conservation professionals' views on governing for coexistence with large carnivores. Biology Conservation, 248, 108668.

[jpe14113-bib-0030] Mattisson, J. , Odden, J. , Nilsen, E. B. , Linnell, J. D. , Persson, J. , & Andrén, H. (2011). Factors affecting Eurasian lynx kill rates on semi‐domestic reindeer in northern Scandinavia: Can ecological research contribute to the development of a fair compensation system? Biological Conservation, 144, 3009–3017.

[jpe14113-bib-0031] Mills, L. S. , Hebblewhite, M. , & Eacker, D. R. (2018). Bayesian population viability analysis for lynx and wolverine in Scandinavia. Swedish Environmental Protection Agency, Report 6793.

[jpe14113-bib-0032] Mitchel, M. S. , Cooley, H. , Gude, J. A. , Kolbe, J. , Nowak, J. J. , Proffitt, K. , Sells, S. N. , & Thompson, M. (2018). Distinguishing values from science in decision making: Setting harvest quotas for mountain lions in Montana. Wildlife Society Bulletin, 42, 13–21.

[jpe14113-bib-0033] Nilsen, E. B. , Brøseth, H. , Odden, J. , & Linnell, J. D. (2012). Quota hunting of Eurasian lynx in Norway: Patterns of hunter selection, hunter efficiency and monitoring accuracy. European Journal of Wildlife Research, 58, 325–333.

[jpe14113-bib-0034] Nilsen, E. B. , Brøseth, H. , Odden, J. , Andrén, H. , & Linnell, J. D. (2011). Prognosemodell for bestanden av gaupe i Norge. NINA Report 774 http://hdl.handle.net/11250/2375639

[jpe14113-bib-0035] Odden, J. , Herfindal, I. , Linnell, J. D. , & Andersen, R. (2008). Vulnerability of domestic sheep to lynx depredation in relation to roe deer density. Journal of Wildlife Management, 72, 276–282.

[jpe14113-bib-0036] Odden, J. , Linnell, J. D. , & Andersen, R. (2006). Diet of Eurasian lynx, *Lynx lynx*, in the boreal forest of southeastern Norway: The relative importance of livestock and hares at low roe deer density. European Journal of Wildlife Research, 52, 237–244.

[jpe14113-bib-0037] Orach, K. , Duit, A. , & Schlüter, M. (2020). Sustainable natural resource governance under interest group competition in policy‐making. Nature Human Behaviour, 4, 1–12. 10.1038/s41562-020-0885-y 32451478

[jpe14113-bib-0038] Pellikka, J. , & Sandström, C. (2011). The role of large carnivore committees in legitimising large carnivore management in Finland and Sweden. Environmental Management, 48, 212–228. 21479919 10.1007/s00267-011-9672-x

[jpe14113-bib-0039] Polasky, S. , Carpenter, S. R. , Folke, C. , & Keeler, B. (2011). Decision‐making under great uncertainty: Environmental management in an era of global change. Trends in Ecology & Evolution, 26, 398–404. 21616553 10.1016/j.tree.2011.04.007

[jpe14113-bib-0040] Raithel, J. D. , Reynolds‐Hogland, M. J. , Koons, D. N. , Carr, P. C. , & Aubry, L. M. (2017). Recreational harvest and incident‐response management reduce human–carnivore conflicts in an anthropogenic landscape. Journal of Applied Ecology, 54, 1552–1562.

[jpe14113-bib-0041] Redpath, S. M. , Linnell, J. D. C. , Festa‐Bianchet, M. , Boitani, L. , Bunnefeld, N. , Dickman, A. , Gutíerrez, R. J. , Irvine, R. J. , Johansson, M. , Majić, A. , McMahon, B. J. , Pooley, S. , Sandström, C. , Sjölander‐Lindqvist, A. , Skogen, K. , Swenson, J. E. , Trouwborst, A. , Young, J. , & Milner‐Gulland, E. J. (2017). Don’t forget to look down–collaborative approaches to predator conservation. Biological Reviews, 92, 2157–2163. 28338282 10.1111/brv.12326

[jpe14113-bib-0042] Redpath, S. M. , Young, J. , Evely, A. , Admas, W. M. , Sutherland, W. J. , Whitehouse, A. , Amar, A. , Lambert, R. A. , Linnell, J. D. C. , Watt, A. , & Gutiérrez, R. J. (2013). Understanding and managing conservation conflicts. Trends in Ecology & Evolution, 28, 100–109. 23040462 10.1016/j.tree.2012.08.021

[jpe14113-bib-0043] Risvoll, C. , Fedreheim, G. E. , & Galafassi, D. (2016). Trade‐offs in pastoral governance in Norway: Challenges for biodiversity and adaptation. Pastoralism, 6, 4.

[jpe14113-bib-0044] Sandström, A. , & Lundmark, C. (2016). Network structure and perceived legitimacy in collaborative wildlife management. Review of Policy Research, 33, 442–462.

[jpe14113-bib-0045] Sandström, C. , Pellikka, J. , Ratamäki, O. , & Sande, A. (2009). Management of large carnivores in Fennoscandia: New patterns of regional participation. Human Dimensions of Wildlife, 14, 37–50.

[jpe14113-bib-0046] Sandström, C. , Sjölander‐Lindqvist, A. , Pellikka, J. , Hiedanpää, J. , Krange, O. , & Skogen, K. (2018). Between politics and management: Governing large carnivores in Fennoscandia. In T. Hovardas (Ed.), Large carnivore conservation and management (pp. 269–290). Routledge.

[jpe14113-bib-0047] Sjölander‐Lindqvist, A. , Risvoll, C. , Kaarhus, R. , Lundberg, A. K. , & Sandström, C. (2020). Knowledge claims and struggles in decentralized large carnivore governance: Insights from Norway and Sweden. Frontiers in Ecology & Evolution, 8, 120.

[jpe14113-bib-0048] Strand, G. H. (2021). The combined effects of centralization and carnivore management on sheep farmers and sheep farming in Norway. Human Dimensions of Wildlife, 26(4), 321–336.

[jpe14113-bib-0049] Swenson, J. E. , & Andrén, H. (2005). A tale of two countries: Large carnivore depredation and compensation schemes in Sweden and Norway. In R. Woodroffe , S. Thirgood , & A. Rabinowitz (Eds.), People and wildlife, conflict or co‐existence? (pp. 323–339). Cambridge University Press.

[jpe14113-bib-0050] Travers, H. , Selinske, M. , Nuno, A. , Serban, A. , Mancini, F. , Barychka, T. , Bush, E. , Rasolofoson, R. A. , Watson, J. E. M. , & Milner‐Gulland, E. J. (2019). A manifesto for predictive conservation. Biological Conservation, 237, 12–18.

[jpe14113-bib-0051] Treves, A. , Chapron, G. , López‐Bao, J. V. , Shoemaker, C. , Goeckner, A. R. , & Bruskotter, J. T. (2017). Predators and the public trust. Biological Reviews, 92, 248–270. 26526656 10.1111/brv.12227 PMC5245106

[jpe14113-bib-0052] Treves, A. , Santiago‐Ávila, F. J. , Popescu, V. D. , Paquet, P. C. , Lynn, W. S. , Darimont, C. T. , & Artelle, K. A. (2019). Trophy hunting: Insufficient evidence. Science, 366, 435–435. 10.1126/science.aaz4389 31649192

[jpe14113-bib-0053] Treves, A. , Wallace, R. B. , & White, S. (2009). Participatory planning of interventions to mitigate human–wildlife conflicts. Conservation Biology, 23, 1577–1587. 19459896 10.1111/j.1523-1739.2009.01242.x

[jpe14113-bib-0054] Tveraa, T. , Stien, A. , Brøseth, H. , & Yoccoz, N. (2014). The role of predation and food limitation on claims for compensation, reindeer demography and population dynamics. Journal of Applied Ecology, 51, 1264–1272. 25558085 10.1111/1365-2664.12322 PMC4279950

[jpe14113-bib-0055] Ullah, I. , & Kim, D. Y. (2020). A model of collaborative governance for community‐based trophy‐hunting programs in developing countries. Perspectives in Ecology and Conservation, 18(3), 145–160.

[jpe14113-bib-0056] van Eeden, L. M. , Crowther, M. S. , Dickman, C. R. , Macdonald, D. W. , Ripple, W. J. , Ritchie, E. G. , & Newsome, T. M. (2018). Managing conflict between large carnivores and livestock. Conservation Biology, 32, 26–34. 28556528 10.1111/cobi.12959

