# Supplementary material for: Quantifying the checks and balances of collaborative governance systems for adaptive carnivore management
Source: J Appl Ecol. 2022 Jan 28;59(4):1038–49. doi: 10.1111/1365-2664.14113 (PMC9306889; doi:10.1111/1365-2664.14113)
Supplement: Supplementary file 1 — Appendix S1 [file JPE-59-1038-s002.pdf]

Appendix S1: Lynx population model structure, fitting and evaluation.

Our population model provides an estimate of the true, region-specific pre-harvest lynx population size at *t* based on the time series of observed number of family groups and harvest bags collected up until *t-1*. It assumes that harvest takes place as an annual pulsed event, enabling us to relate for region *k* the pre-harvest population size in year *t-1*, $N_{t-1,k}$, to the post-harvest population size $X_{t-1,k}$ by removing the observed harvest $H_{t-1,k}$:

$$X_{t-1,k} = N_{t-1,k} - H_{t-1,k} \qquad [1].$$

Following the harvest of lynx in February and March, breeding occurs in spring and birth in early summer. Consequently, population growth $\lambda_{t-1,k}$ between $X_{t-1,k}$ and the pre-harvest population at *t* can be written as

$$N_{t,k} = X_{t-1,k} * \lambda_{t-1,k} \qquad [2].$$

We assume $\log(\lambda_{t-1,k})$ to be a random sample from a normal distribution with mean $\log(\bar{\lambda}_k)$ and variance σ_e^2, simulating environmental stochasticity due to interannual variation in recruitment and non-harvest mortality:

$$\log(\lambda_{t-1,k}) \sim Norm(\log(\bar{\lambda}_k), \sigma_e^2) \qquad [3].$$

To relate the estimated (true) pre-harvest population size $N_{t-1,k}$ to the annual monitoring data $FG_{t-1,k}^{observed}$, we first have to account for the fact that only observations of family groups $FG_{t-1,k}^{observed}$ (females with kittens) are included in the monitoring scheme. To convert the pre-harvest population size $N_{t-1,k}$ to a predicted number of family groups $FG_{t-1,k}^{predict}$, we assume the following relationship:

$$FG_{t-1,k}^{predict} = \frac{N_{t-1,k}}{\beta_k} \qquad [4],$$

in which *βk* is the inverse proportion of family groups in the lynx population in region *k* (33). We use an informative prior for *β* based on previous studies of lynx demography in Scandinavia (Andrén et al., 2020). To account for measurement error in the monitoring data, we assume that the predicted number of family groups $FG_{t-1,k}^{predict}$ is related to the observed number of family groups $FG_{t-1,k}^{observed}$ as follows:

$$FG_{t-1,k}^{observed} = FG_{t-1,k}^{predict} + \varepsilon_{t-1} \qquad [5],$$

$$\varepsilon_{t-1} \sim Norm(0, \sigma_m^2) \qquad [6].$$

Using this model, we generated predictions of the number of lynx for each region and year *t* between 2012 and 2018, representing the period during which the model was available to the regional Secretariats. number of lynx More specifically, for each year *t*, we fitted the model to lynx count and harvest data collected between 1996 (when lynx monitoring began; Dataset S1 in SI Appendix) and *t-1*, mimicking the process used by the Secretariats to generate year on year predictions of N_t for their respective regions. The model also enabled estimates of the conversion factor β_k and of the mean population growth rate ($\bar{\lambda}_k$) to be derived at a regional level, the latter with associated standard deviation sd($\bar{\lambda}_k$). As with predictions of N_t, new estimates of $\bar{\lambda}_k$ and β_k were generated for every run of the model between 2012 and 2018, thus mimicking its use by regional Secretariats.

Model-fitting was carried out using the R2jags package in R (Su & Yajima, 2015). For each model run, we ran three chains of 1,500,000 iterations following a 750,000 burn-in. Model convergence was assessed from both the R-hat value (<1.1 for reliable convergence) and from a visual inspection of chain trace plots (Gelman & Rubin, 1992). Model-based predictions of N_t that were deemed to be unreliable (R-hat value consistently > 1.1) were replaced by the observed lynx count at *t-1*.

References

Gelman, A., & Rubin, D. B. (1992). Inference from iterative simulation using multiple sequences. *Statistical Science, 7*, 457–472.

Su, Y-S., & Yajima, M. (2015). R2jags: Using R to Run 'JAGS'. *R package version 0.5-7*, https://CRAN.R-project.org/package=R2jags
