# Supplementary material for: Quantifying the checks and balances of collaborative governance systems for adaptive carnivore management
Source: J Appl Ecol. 2022 Jan 28;59(4):1038–49. doi: 10.1111/1365-2664.14113 (PMC9306889; doi:10.1111/1365-2664.14113)
Supplement: Supplementary file 2 — Appendix S2 [file JPE-59-1038-s001.pdf]

Appendix 2. Figures S2-1 to S2-4, Tables S2-1 and S2-2.

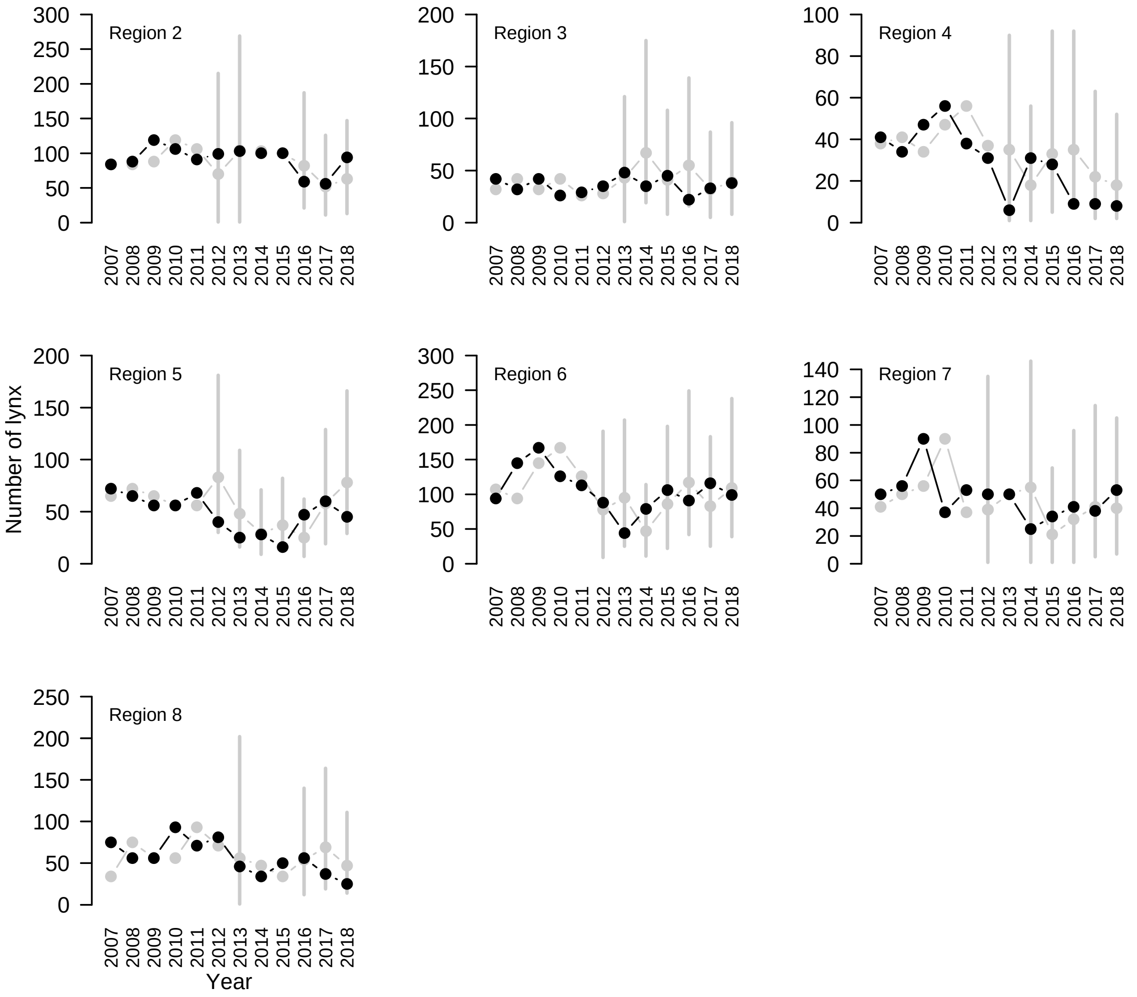

Figure S2-1. Time series of observed and estimated number of lynx (full black and grey circles, respectively) between 2007 and 2018 for the seven Norwegian management regions considered. Estimated numbers of lynx and their associated 95% confidence intervals (grey brackets) are derived from a state-space population model that estimates true, region-specific pre-harvest lynx population size and population growth rate from the observed number of reproductive females and recorded harvest.

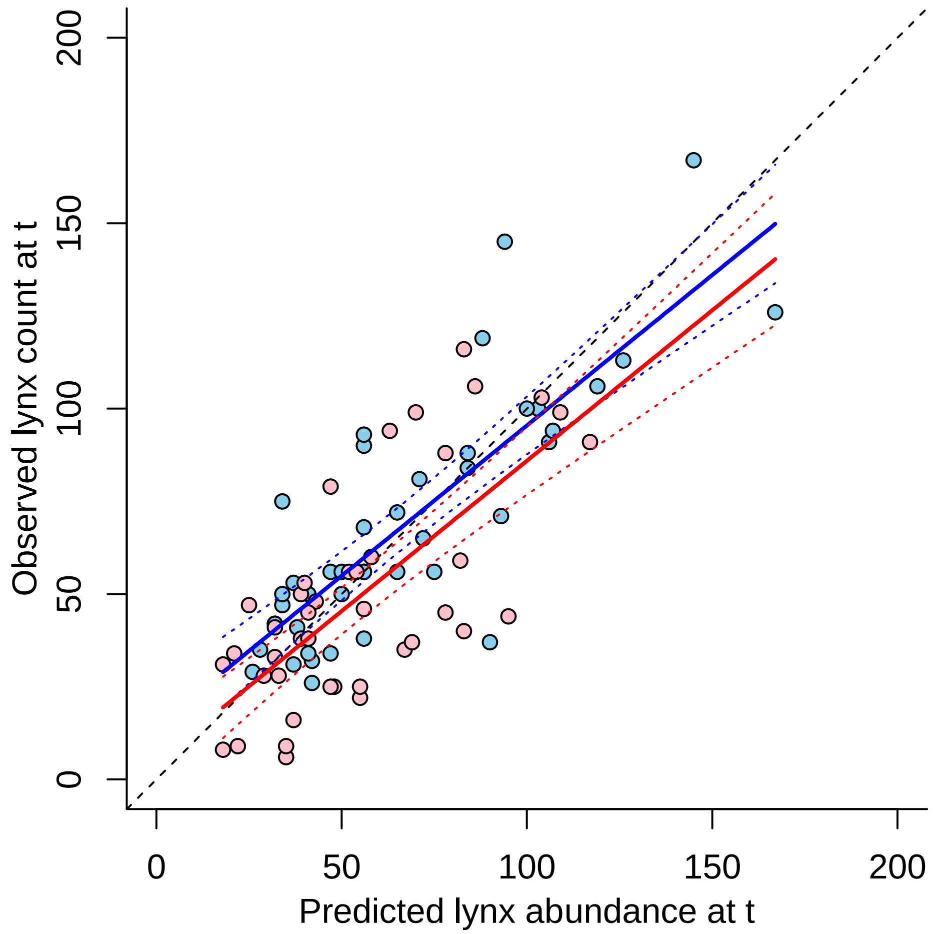

Figure S2-2. Relationship between observed and predicted values of regional lynx abundance at time *t* between 2007 and 2018. Blue and red filled circles relate to predictions of N_t that were generated using the count- and model-based approaches, respectively. Full blue and red lines represent separate relationships estimated for count- and model-based approaches from a linear model with lynx prediction and prediction approach as additive effects. Dotted lines denote 95% confidence intervals associated with the fitted lines. The dashed line represents the x=y line. The count-based approach involves taking the value of N_{t-1} as a prediction for N_t. The model-based approach involves the application of a state-space population model to predict the true, region-specific pre-harvest lynx population size at *t* based on the time series of observed number of family groups and harvest bags collected up until *t-1*

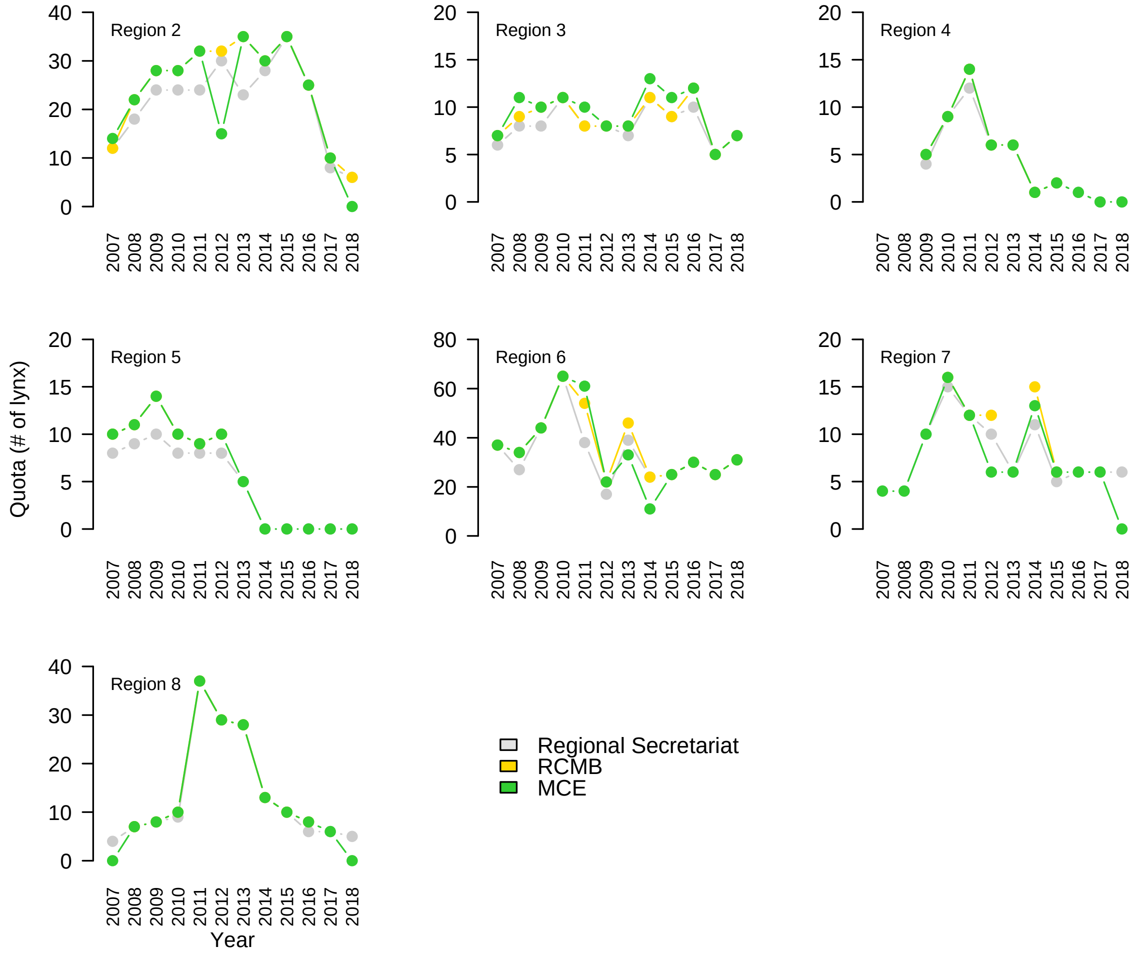

Figure S2-3. Time series of lynx quota decisions taken by the regional Secretariats, the Regional Carnivore Management Boards (RCMBs) and the Ministry of Climate and Environment (MCE) between 2007 and 2018 in seven Norwegian carnivore management regions.

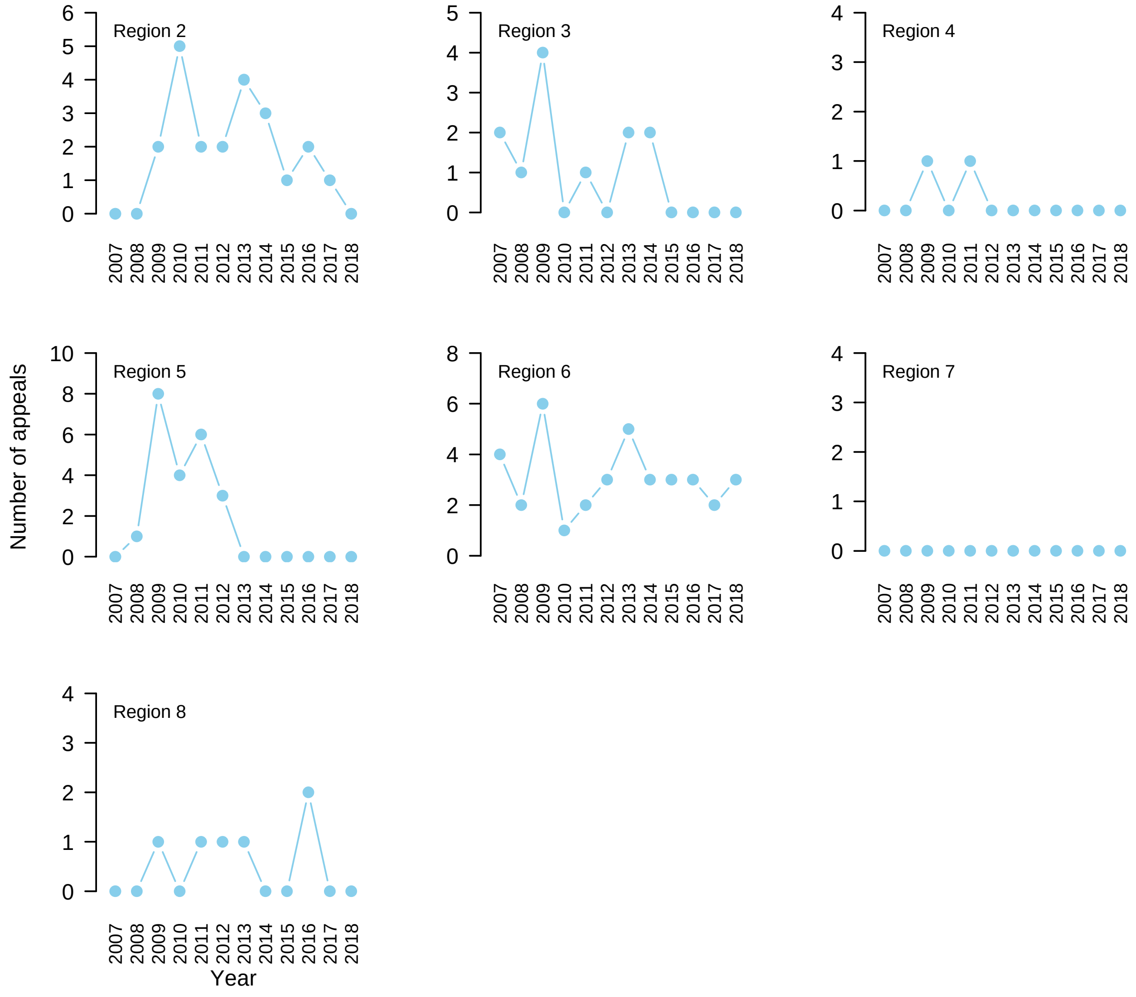

Figure S2-4. Time series of the number of appeals relating to lynx harvest recorded between 2007 and 2018 in seven Norwegian carnivore management regions.

Table S2-1. Model comparison outputs for the Secretariat, Regional Carnivore Management Board (RCMB) and Ministry of Climate and Environment (MCE) lynx quota decision stages, as well as for the appeal process. Grey highlights indicate models used for inference and predictions.

Decision stage	Decision step	*n*	Fixed effects structure	df	Log-likelihood	AICc	Weight
Regional Secretariat	-	82	~ PTR * Region	16	-248.3	537.0	0.92
			~ PTR + Region	10	-259.4	541.9	0.08
			~ Region	9	-277.7	576.0	0
			~ PTR	4	-299.9	608.3	0
			~ 1	3	-322.7	651.8	0
RCMB	Probability of quota change	63	~ Region	8	-34.9	88.4	0.64
			~ PTR + Region	9	-34.8	90.9	0.18
			~ 1	2	-43.7	91.5	0.14
			~ PTR	3	-43.7	93.7	0.05
			~ PTR * Region	15	-33.4	107.0	0
	Magnitude of change	31	~ PTR	4	-66.0	141.4	0.96
			~ 1	3	-70.4	147.7	0.04
Appeal	Probability of appeal	82	~ PTR	3	-31.8	70.0	0.86
			~ PTR + Region	7	-28.8	73.8	0.13
			~ Region	6	-33.2	80.1	0.01
			~ 1	2	-38.8	81.7	0
			~ PTR * Region	11	-28.0	83.8	0
	Number of appeals	39	~ Region	8	-63.8	148.5	0.44
			~ 1	3	-71.1	148.8	0.36
			~ PTR	4	-73.1	153.3	0.11
			~ PTR + Region	9	-66.7	153.7	0.09
			~ PTR * Region	14	-62.4	170.3	0
MCE	Probability of quota change	82	~ PTR	3	-34.8	76.0	0.52
			~ PTR + # appeals	4	-34.2	77.9	0.20
			~ 1	2	-37.5	79.1	0.10
			~ # appeals	3	-37.5	81.3	0.09
			~ PTR + Region	7	-33.4	82.9	0.09
			~ Region + PTR + # appeals	8	-33.2	85.2	0
			~ Region	6	-36.5	86.5	0
			~ # appeals + Region	7	-36.5	89.1	0
	Magnitude of quota change	15	~ PTR + # appeals	5	-39.1	94.9	1
			~ PTR	4	-47.1	106.3	0
			~ # appeals	4	-47.4	106.7	0
			~ 1	3	-49.4	107.0	0

Table S2-2. Summary of regional lynx population sizes (*N*) prior to 2018 and additional parameters – including the mean yearly population growth rate ($\bar{\boldsymbol{\lambda}}$) and associated standard deviation, the family group to lynx number conversion factor $\boldsymbol{\beta}$, and the regional target – used to forecast lynx population dynamics between 2019 and 2030.

	N estimate															
Region	2007	2008	2009	2010	2011	2012	2013	2014	2015	2016	2017	2018	Regional $\bar{\boldsymbol{\lambda}}$	sd($\bar{\boldsymbol{\lambda}}$)	β	Regional target
2	85	93	116	106	93	99	103	101	97	64	59	90	1.265	0.112	0.159	75
3	39	36	37	31	32	33	42	38	38	28	31	36	1.301	0.144	0.156	32
4	39	38	45	50	38	26	18	21	19	13	11	12	1.192	0.131	0.159	38
5	70	65	57	56	63	40	28	26	23	43	55	47	1.188	0.111	1.160	62
6	102	141	161	130	111	80	57	75	102	96	111	95	1.248	0.108	0.156	77
7	48	58	82	49	51	48	46	35	33	38	40	49	1.120	0.123	0.160	62
8	69	57	59	90	74	78	49	38	47	51	37	28	1.194	0.126	0.161	62
